# Identification of ABC Transporter Interaction of a Novel Cyanoquinoline Radiotracer and Implications for Tumour Imaging by Positron Emission Tomography

**DOI:** 10.1371/journal.pone.0161427

**Published:** 2016-08-23

**Authors:** Rozanna L. Slade, Federica Pisaneschi, Quang-De Nguyen, Graham Smith, Laurence Carroll, Alice Beckley, Maciej A. Kaliszczak, Eric O. Aboagye

**Affiliations:** Comprehensive Cancer Imaging Centre, Imperial College London, Faculty of Medicine, Hammersmith Hospital Campus, Du Cane Road, London, W12 0NN, United Kingdom; Wayne State University, UNITED STATES

## Abstract

**Background:**

The epidermal growth factor receptor (EGFR) is overexpressed in many cancers including lung, ovarian, breast, head and neck and brain. Mutation of this receptor has been shown to play a crucial role in the response of non-small cell lung carcinoma (NSCLC) to EGFR-targeted therapies. It is envisaged that imaging of EGFR using positron emission tomography (PET) could aid in selection of patients for treatment with novel inhibitors. We recognised multi-drug resistant phenotype as a threat to development of successful imaging agents. In this report, we describe discovery of a novel cyanoquinoline radiotracer that lacks ABC transporter activity.

**Methods:**

Cellular retention of the prototype cyanoquinoline [^18^F](2*E*)-N-{4-[(3-chloro-4-fluorophenyl)amino]-3-cyano-7-ethoxyquinolin-6-yl}-4-({[1-(2-fluoroethyl)-1H-1,2,3-triazol-4-yl]methyl}amino)-but-2-enamide ([^18^F]FED6) and [^18^F](2*E*)-N-{4-[(3-chloro-4-fluorophenyl)amino]-3-cyano-7-ethoxyquinolin-6-yl}-4-[({1-[(2*R*,5*S*)-3-fluoro-4,5-dihydroxy-6-(hydroxymethyl)oxan-2-yl]-1*H*-1,2,3-triazol-4-yl}methyl)amino]but-2-enamide ([^18^F]FED20) were evaluated to establish potential for imaging specificity. The substrate specificity of a number of cyanoquinolines towards ABC transporters was investigated in cell lines proficient or deficient in ABCB1 or ABCG2.

**Results:**

FED6 demonstrated substrate specificity for both ABCG2 and ABCB1, a property that was not observed for all cyanoquinolines tested, suggesting scope for designing novel probes. ABC transporter activity was confirmed by attenuating the activity of transporters with drug inhibitors or siRNA. We synthesized a more hydrophilic compound [^18^F]FED20 to overcome ABC transporter activity. FED20 lacked substrate specificity for both ABCB1 and ABCG2, and maintained a strong affinity for EGFR. Furthermore, FED20 showed higher inhibitory affinity for active mutant EGFR versus wild-type or resistant mutant EGFR; this property resulted in higher [^18^F]FED20 cellular retention in active mutant EGFR expressing NSCLC.

**Conclusion:**

[^18^F]FED20 binds EGFR but is devoid of ABC transporter activity, thus, has potential for EGFR imaging.

## Introduction

The epidermal growth factor receptor (EGFR) is a 170 kDA transmembrane tyrosine kinase receptor member of the ErbB family of receptors, which includes HER2 (ErbB2), HER3 (ErbB3) and HER4 (ErbB4) [1]. Pathological expression of EGFR has been observed in many cancers including head and neck, breast, pancreatic, non-small cell lung cancer (NSCLC) and colorectal cancers [2]. Currently clinically approved therapies which target EGFR include monoclonal antibodies such as cetuximab or panitumumab [3,4], and small molecular tyrosine kinase inhibitors (TKI) such as gefitinib and erlotinib [4]. In the context of NSCLC, the efficacy of TKI’s was found to be significantly higher in patients who express active mutant EGFR of which the L588R and Del E746-A750 mutations are the most common. The frequency of mutations in EGFR recorded in NSCLC patients has been found to vary [5–7], but can be as high as 44% in female Asian patients with adenocarcinoma and no previous history of smoking [5,8,9]. The Iressa Pan-Asia-Study (IPASS), which compared gefitinib to a standard carboplatin and paclitaxel chemotherapy in a first line treatment setting in NSCLC Asian patients [10] was the first study that demonstrated a benefit of TKI therapy in selected NSCLC patients. The most prevalent mechanism of acquired resistance is a secondary mutation in EGFR (T790M); this mutation occurs in half of patients administered reversible TKIs and results from an increase in affinity for the receptor for ATP [11,12]. The issue of acquired resistance is complex; more recently C797S mutations were reported as a new mechanism to third generation TKIs [13]. Despite advances in EGFR targeted therapies and the increasing number of EGFR inhibitors undergoing preclinical evaluation, there are currently no non-invasive probes for the *in vivo* imaging of EGFR used routinely in the clinical setting. Such probes could be used to aid patient stratification in a setting where multiple biopsies are difficult to obtain.

A number of positron emission tomography (PET) probes targeting EGFR have been developed and preclinically evaluated in the last decade. Both gefitinib and erlotinib have been radiolabelled with carbon-11 and/or fluorine-18. [^18^F]Gefitinib gave disappointing results as no differences were detected *in vitro* or *in vivo* in models that expressed different levels of EGFR or mutant forms of EGFR [14]. On the other hand [^11^C]erlotinib showed more promising results *in vivo*, with higher uptake in active mutant EGFR compared to wild type EGFR expressing xenografts [15]. Furthermore, a clinical trial evaluating the use of [^11^C]erlotinib for the *in vivo* assessment of EGFR mutational status in NSCLC patients have been published [16]. This small trial of five patients with del E746-A750 and five patients with WT EGFR showed that the volume of distribution of the radiotracer was on average two times greater in the del E746-A750 EGFR than in the wild-type EGFR expressing tumours [16].

**Fig 1:**
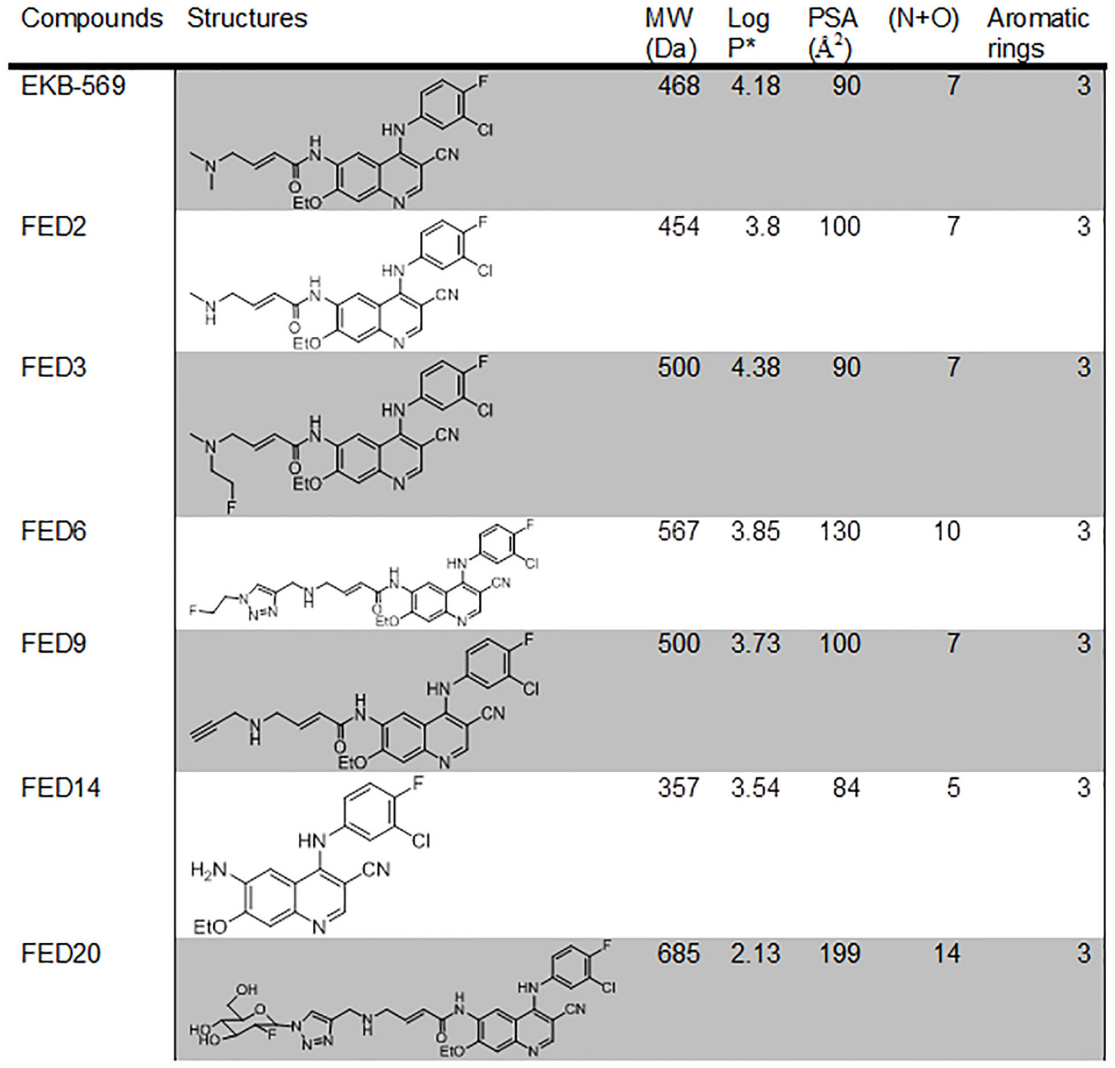
Physicochemical properties of cyanoquinoline compounds. Molecular weight (MW), the sum of oxygen and nitrogen atoms (N+O), calculated Log P (Marvin Sketch; https://www.chemaxon.com/products/marvin/marvinsketch), and polar surface area (PSA). LogarIthmic value of acid dissociation constant (pKA).

Other EGFR targeting radiotracers have been developed based on irreversible TKIs, which covalently bind cysteine-773 of the EGFR tyrosine kinase-binding site. Irreversible tracers were believed to be more appropriate for *in vivo* imaging as they would compete less with the high intracellular ATP concentration for domain occupancy [17]. One of the first compounds of this series was [^18^F]ML04, which although retained selectivity for EGFR *in vitro* was poorly specific *in vivo*, with high uptake in EGFR negative tumour models and no decrease in tumour radioactivity upon pre-treatment with a blocking dose of cold ML04 [17]. Our group developed fluorine-18 labelled cyanoquinoline irreversible probes including FED6 ([^18^F](2E)-N-{4-[(3-chloro-4-fluorophenyl)amino]-3-cyano-7-ethoxyquinolin-6-yl}-4-({[1-(2-fluoroethyl)-1H-1,2,3-triazol-4-yl]methyl}amino)-but-2-enamide; Fig 1) [18]. FED6 showed high uptake in EGFR overexpressing tumours compared to low EGFR expressing tumours and was metabolically stable. There were however high levels of uptake in the intestines. This was partially attributed to less favourable pharmacokinetics and/or non-specific interactions. In the context of PET imaging of mutant forms of EGFR, one of the first examples of irreversible TKI reported in the literature is [^18^F]PEG6-IPQA; [^18^F]PEG6-IPQA was shown to bind with higher selectivity and specificity to L858R activating mutant EGFR expressing tumour xenografts compared to resistant EGFR L858R/T790M mutant xenografts [19]. More recently, Slobbe and co-workers have demonstrated potential usefulness of [^18^F]afatinib as an irreversible TKI for imaging treatment-sensitive xenografts harboring exon-19 deletion mutations in EGFR [20].

One of the threats to use of quinoline or quinazoline-based molecules as imaging agents is susceptibility to efflux by members of the ATP binding cassette transporter proteins (ABC transporters) overexpressed at the cell surface of most tumours [21]. The key ABC transporter family members of relevance to the multi-drug resistance (MDR) phenotype in oncology include permeability glycoprotein (P-gp also known as ABCB1), breast cancer resistance protein (BCRP or ABCG2) and multidrug resistance protein 1 (MRP1or ABCC1) [21,22]. In relation to probe design, compounds that are substrates for ABCB1 despite having different structures share certain physicochemical properties such as a molecular weight (MW) > 400 Da, log*P* > 3 and polar surface area (PSA) > 85 Å^2^ [23]. Both gefitinib and erlotinib have been found to be substrates of ABCB1 and ABCG2 [24,25]; furthermore, gefitinib has also been shown to be an inhibitor of ABCG2 [22]. Second generation irreversible inhibitors including EKB-569 (pelitinib; Fig 1) from which the cyanoquinoline series of compound was based, also interact with ABCG2 [24]. Regarding EGFR targeted radiotracers, [^18^F]ML04 was found to be a substrate for ABCB1 [17]; more recently [^18^F]afatinib was also shown to be responsive to ABCB1 modulation [20]. The threat posed by ABC substrate specificity for imaging probes results from the low mass of injected compound resulting in efficient efflux and consequently low tumour localisation. Against this background, we investigated the interaction of a series of cyanoquinolines EGFR antagonists previously evaluated in our laboratory for their substrate specificity for key ABC transporters, ABCB1 and ABCG2. Based on the findings, we tested a new probe and found it to be devoid of ABC transporter substrate specificity.

## Materials and Methods

### Inhibitors and Cell Lines

Unless otherwise stated, all compounds were purchased from Sigma (St Louis, Missouri, USA). For brevity, cell lines used to investigate ABC transporter specificity, together with their respective growth media are summarised in Table 1. 3T3 and 3T3 transfected with cDNA expressing P-gp (pHamdr1) were kindly provided by Dr E Schuetz from St Jude’s Children Research Hospital (Memphis, TN, USA) [26], and MCF7 and mitoxantrone (MX)-resistant subclones MCF7MX by Dr E Schneider from the University of Maryland (Baltimore, MD, USA) [27]. Generation of the isogenic PC9ER from PC9 lung cancer cells has been reported [28]. Media were supplemented with 1% penicillin/streptomycin and 10% fetal bovine serum (FBS) (all purchased from Invitrogen, Carlsbad, California, USA). All cells were grown in a humidified atmosphere at 37°C and 5% CO_2_.

**Table 1:** Summary of cell lines used.

Cell line	Source	EGFR mutation status	Other genes	Growth media
H1650	ATCC	Del E746-A750	PTEN null	RPMI
H358	ATCC	WT		RPMI
A549	ATCC	WT	k-RAS mutant	RPMI
H3255	ATCC	L858R		RPMI
H1975	ATCC	L858R/T790M		RPMI
PC9	Dr Olivier Pardo, Imperial College London, UK	Del E746-A750		RPMI
PC9ER	Dr Olivier Pardo, Imperial College London, UK	Del E746-A750/T790M		RPMI
A431	ATCC	WT		DMEM
MCF7	Dr E. Schneider, University of Maryland, USA	WT		DMEM
MCF7MX	Dr E. Schneider, University of Maryland, USA	WT	ABCG2	DMEM
3T3	Dr E. Schuetz, TN USA	WT		DMEM
3T3-MDR1	Dr E. Schuetz, TN USA	WT	MDR1	DMEM

WT, wildtype; RPMI, Roswell Park Memorial Institute medium-1640; DMEM, Dulbecco's Modified Eagle Medium.

### Inhibition of EGFR Autophosphorylation

Affinity of the cyanoquinolines for EGFR was established by measuring their inhibition of EGFR autophosphorylation via western blot. Cells were seeded in 6 well plates and following 24 h of growth, were subject to overnight serum starvation. The cells were then treated with increasing concentrations of cyanoquinolines prepared in serum free growth media from a DMSO stock (final DMSO concentration < 1%) for 3 h at 37°C, and stimulated for receptor autophosphorylation by addition of 100 ng/mL of EGF during the last 15 minutes of drug incubation. After 3 h the cells were placed on ice, media removed and then the cells were washed two times with ice cold phosphate buffered saline (PBS). Lysates were analysed by western blot to measure changes to p-EGFR Y1068.

### Western Blot Analysis

Cell lysates were prepared by addition of radioimmunoprecipitation assay (RIPA) buffer (50mM Tris-HCl pH 7.2, 150mM NaCl, 1% NP40, 1% sodium deoxycholate, 0.1% sodium dodecyl sulfate) supplemented with 1 x protease and phosphatase inhibitor cocktail (1mM 4-(2-Aminoethyl)benzenesulfonylfluoride hydrochloride, 800 nM aprotinin, 50 μM bestatin, 15 μM E-64, 5μM EDTA, 20 μM leupeptin, 10 μM pepstatin A; Thermo scientific, Waltham, Massachusetts, USA). Cells were collected into a microcentrifuge tube and sonicated for 30 seconds on ice. Cellular protein levels were assessed using a bicinchoninic acid (BCA) protein assay kit according to the manufacturer’s instructions (Thermo scientific). Lysates containing 10 x reducing agent (Invitrogen) and 4x NuPAGE^®^ LDS Sample Buffer (4X) (lithium dodecyl sulphate) (Invitrogen) were denatured by heating for 10 minutes at 70°C; 15 μg of the protein samples were separated using 4–15% polyacrylamide Mini-PROTEAN TGX precast gels (Bio-Rad, Hemel Hempstead, UK). Samples were transferred to polyvinylidene difluoride (PVDF) membrane using Trans-Blot Turbo system BIO-RAD (Hercules, California, USA). Membranes were blocked with 5% milk or 5% bovine serum albumin (BSA) in Tris-buffered saline-tween 20 (TBST; 50 mM Tris, 150 nM NaCl, 0.05% tween 20) for 1 h at room temperature. The membranes were then incubated overnight with primary antibody prepared in 1% milk or 1% BSA in TBST. Primary antibody (antibody, source, catalog number, antibody dilution): p-EGFR-Y1068, Cell Signaling Technology, 2234, 1/1000; EGFR, Cell Signaling Technology, 2232, 1/2000; ABCG2, Abcam, ab3380, 1/1000; ABCB1, Abcam, ab129450, 1/1000; b-actin, Sigma, SAB5500001, 1/5000. After overnight incubation, membranes were washed three times for 10 minutes with TBST before incubating with secondary goat anti-rabbit (Sc-2004) or goat anti-mouse (Sc-2005) antibody (Santa Cruz Biotechnology, Dallas, Texas, USA) at 1/2000 dilution for 1 h at room temperature. Membranes were washed a further three times for 10 minutes with TBST. Blots were visualised using chemoluminescence (ECL, GE Healthcare, Little Chalfont, UK) and developed on Amersham Hyperfilm ECL (GE Healthcare). β-Actin was used as a loading control.

### Cell Viability Assay

Cell viability by the sulforhodamine B (SRB) assay was used to assess cell growth and cytotoxicity of test compounds [29]. Cells were seeded at densities of 3,500 to 5,000 cells per well in 96 well plates overnight and pre-treated for 1 h with ABC transporter modulators: 10 μM fumitremorgin C (FTC; in MCF7MX and MCF7 cells), or 0.3 μM of zosuquidar (in 3T3 and 3T3-MDR1). Cells were then treated with test compounds for 72 h, at 37°C. After drug treatment, cells were fixed by addition of 50% trichloroacetic acid (TCA), and the plates washed and dried to allow staining with 0.4% solution of sulforhodamine B (Sigma) in 1% acetic acid for 30 minutes. Excess dye was removed upon washing with 1% acetic acid. Plates were dried and bound protein was solubilised upon addition of 150 μL of 10 mM Tris base buffer. Absorbance was measured at 540 nm using Thermo Multiskan-Ex plate reader. Cell viability versus compound concentration curves were plotted as a percentage of control cells. The concentration of drug that gave 50% inhibition in cell growth (GI50) was extrapolated from these curves using the sigmoid curve fit with variable slope in Prism (GraphPad Prism version 5.01, GraphPad Software, San Diego California USA). Three to six replicate wells were used for each drug concentration and experiments were carried out on three separate occasions for each compound.

### Caco2 Transwell Assay

The caco2 transwell assay was performed to assess permeability. Cells were seeded at a density of 60,000 cells per well in 24 well transwell plates (Millipore, Billerica, Massachutsetts, USA), and maintained in DMEM supplemented with 4.5 g/mL glucose (20% FBS, 1% penicillin/streptomycin, 1% glutamine) for 21 days at 37°C in 5% CO_2_. After 21 days the cells differentiate into enterocytes and express all three MDR proteins, ABCB1 and ABCG2 at the apical side and ABCC1 on the basal side. On day 21, cells were washed three times with Hank's Balanced Salt Solution (HBSS) (Fisher scientific, Loughborough, UK) and the trans-epithelial electric resistance (TERR) was measured using an Epithelial Voltohmmeter (EVOM) (World Precision Instruments, Sarasota, USA); only transwells with TERR of 400 Ω/cm^2^ or above were used for the experiment. Cells were treated either at the apical (n = 3) or basal (n = 3) side of the transwell by addition of 10 μM of cyanoquinoline compound or 50 μM vinblastine prepared in a volume of 400 μL HBSS (compound added to apical well) or 800 μL of HBSS (added to basal well). When transport inhibitors FTC (10 μM) or zosuquidar (0.3 μM) were used, they were added to both the apical and basal wells 1 h prior to drug treatment. After 1 h pre-treatment the medium was removed and cells were treated with the compounds prepared in HBSS containing FTC or Zosuquidar.

Plates were incubated in shaker incubator at 37°C and 60 *rpm* for 120 min. After incubation, 200 μL aliquots from the apical and the basal wells were collected and analysed using high performance liquid chromatography (HPLC). Samples were run on a Millipore Waters HPLC system which included a 717 plus autosampler and 2487 dual wavelength absorbance detector system. The stationary phase comprised of a Phenomenex Luna C_18_ reverse phase column (150 X 4.6 mm; 5 μm particle size). The mobile phase comprised water (0.1% TFA) and acetonitrile (0.1% TFA) (80:20 v/v) at a flow rate of 1 mL/min for all compounds except vinblastine. For vinblastine, the stationary phase comprised a Supelcosil LC-ABZ reverse phase column (5 × 4.6 mm; 5 μm particle size). The mobile phase comprised water (0.1% formic acid) and methanol (90:10 to 10:90 v/v gradient over 20 min). Peak areas corresponding to the compounds of interest were recorded. Using standard curves the areas under the curve were converted into concentrations of compound. The permeability coefficient (Papp) was calculated using the following equation [30]: Papp = (dQ/dt)/A C0, where A is the surface area of the transwell membrane in cm^2^ (= 0.33 cm^2^), C0 is the drug concentration in the donor chamber at time t = 0 and dQ/dt is the rate of transfer of the compound to the receiver chamber, determined from the slope of the graph concentration (dQ) versus time (dt). Papp values corresponding to absorption and secretion were assessed and used to calculate efflux ratio.

$$Efflux\;ratio\;=\frac{\left(\text{Papp B}-\text{A}\right)}{\left(\text{Papp A}-\text{B}\right)}$$

Where Papp A-B = Papp absorption and Papp B-A = Papp secretion.

The integrity of the caco2 cell membrane was assessed for each well following the experiment by addition of a 100 μM solution of luciferase yellow (Sigma) in HBSS to the apical side of the plate. The plates were placed in the shaker incubator for 1 h at 37°C and 60 *rpm*. Samples from the basal wells were transferred into a 96 well plate and the levels of luciferase yellow were assessed by measuring with an excitation of 450 nm and an emission of 540 nm using the PerkinElmer Wallac 1420 counter. The membrane was considered intact if measured values from the basal side were inferior to 1% that of the measured values on the apical side.

### siRNA Transfection

SiRNA transfections were carried out using ON TARGET plus-SMARTpool siRNA or non-targeting siRNA (Dharmacon, Lafayette, Louisianna, USA); ABCG2, L-009924-00-0005 and scramble (non-targeting) D-001810-10-05. Lipofectamine RNAiMAX (Invitrogen UK) was used as the transfection reagent and was prepared as indicated on the product protocol. Cells were seeded in black 96 well plates in antibiotic free media. On the day of transfection, siRNA and Lipofectamine were separately diluted in opti-MEM media, mixed in a 1 to 1 ratio and incubated at room temperature for 20 min; the siRNA-Lipofectamine complexes were then added to the cells. Fresh media containing 1% penicillin streptomycin was added to the cells after 24 h followed by Hoechst fluorescent assays at 72 h.

### Cell Uptake Assay

[^18^F]FED6 and [^18^F]FED20 were synthesised as previously reported [18,31]. To increase specific radioactivity of [^18^F]FED20, we modified the synthesis by decreasing the amount of alkyne precursor from 1 mg down to 0.5 mg. While this had the desired effect of increasing the specific activity from 7.3 GBq/μmol up to 52 GBq/μmol, the conversion of the alkyne to the triazole product dropped from > 95% down to 45%. Cells were seeded in 6 well plates and [^18^F]FED6 (0.22 MBq) or [^18^F]FED20 (0.55 MBq or 0.74 MBq) added to each well and incubated at 37°C for 1 h. Media were then removed and cells were washed twice with PBS. The cells were trypsinised and centrifuged for 3 minutes at 2000 *rpm* to obtain a pellet. The cell pellet was washed once with PBS and resuspended in 200 μL of RIPA buffer. Cell lysates were transferred to gamma counting tubes and the radioactivity in the pellet was measured on a Packard Cobra II gamma counter, (Perkin-Elmer, Waltham, Massachusetts, USA). Protein concentrations of samples were evaluated using Pierce^®^ BCA Protein Assay Kit (Thermo Scientific, Waltham, Massachusetts, USA). Data were expressed as background- and decay-corrected counts per minute (CCPMA) per μg of cellular protein.

### Statistics

Unless otherwise specified, data were expressed as mean ± standard error (SE). Data were compared by unpaired two tailed Student’s t-test using GraphPad Prism. Statistical significance was assigned to values ≤ 0.05. * corresponded to p value < 0.05 and ** corresponded to p < 0.01.

## Results

### Cyanoquinoline Compound FED6 Is a Substrate for ABCB1 and ABCG2

As shown in Fig 1, the series of FED compounds possess several of the physico-chemical properties which are associated with ABCB1 and ABCG2 substrate specificity (MW > 400 Da (567), PSA > 85Å^2^ (130 Å^2^), number of nitrogen and oxygen atoms (N+O) > 8 (10), number of aromatic rings > 2 (3) and lipophilicity (Log*P*) < 1 or > 5 (3.85)). The exception to this was FED14 (synthetic intermediate molecule in the series) [23].

**Fig 2:**
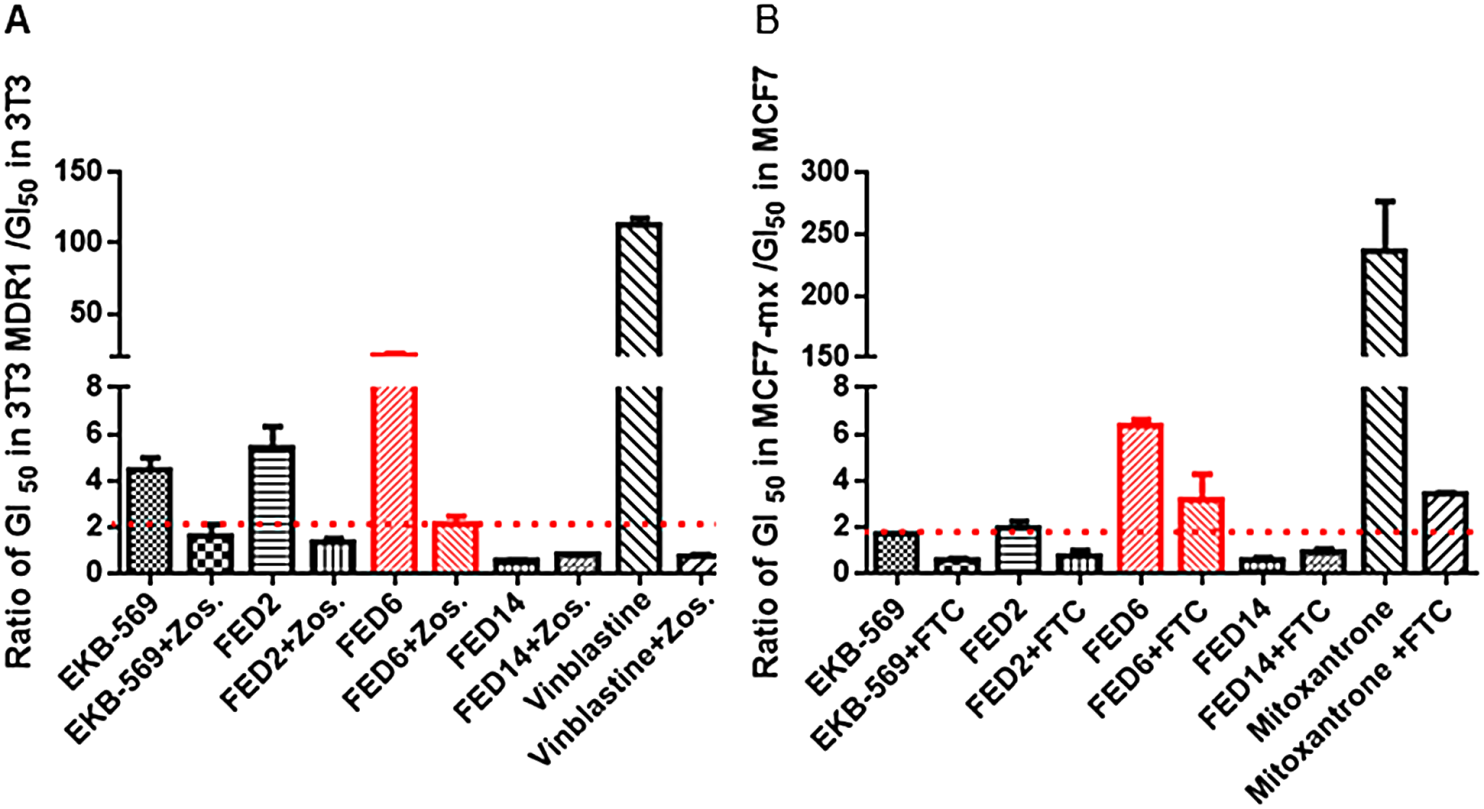
Growth inhibitory effect associated with a series of cyanoquinoline molecules in 3T3, 3T3-MDR1, MCF7, and MCF7MX cells. A. Ratios of GI50 values in 3T3-MDR1 over GI50 values in 3T3 cells with and without pre-treatment with zosuquidar (zos). B. Ratios of GI50 values in MCF7MX over GI50 values in MCF7 cells with and without pre-treatment with fumitremorgin C (FTC). Data indicate mean ratios ± SE of ratios obtained from three separate experiments.

We investigated the substrate specificity of the cyanoquinoline series to the two ABC transporters previously reported to interact with TKI compounds: ABCB1 and ABCG2 by a number of approaches. First, we used two paired cell models - 3T3 cells (transfected with an empty vector) and 3T3-MDR1 cells (transfected with the human MDR1 gene which encodes ABCB1) or MCF7 cells and a mitoxantrone resistant, MCF7MX cell line, which overexpresses ABCG2—and investigated the impact of FED6 treatment on cell viability. The GI50 (causing 50% growth inhibition) values obtained from these cell viability assays are summarised in Table 2. EKB-569, FED2 and FED6 were associated with GI50s of 5.75, 36, and > 100 μM, respectively, in the 3T3-MDR1 cells; corresponding values in the 3T3 cells were 1.06, 7 and 10.7 μM. Compounds associated with ABC transporter proficient to isogenic ABC transporter deficient cell line GI50 ratio of > 2 are considered to be actively effluxed by the transporters. There was a 5.4, 5.1 and > 10 fold increase in GI50 values for EKB-569, FED2 and FED6, respectively, in the ABCB1 overexpressing cell line (Fig 2A) suggesting that all three compounds were effluxed via ABCB1. Pre-treatment with specific ABCB1 inhibitor, zosuquidar [32], led to a decrease in the GI50 of EKB-569, FED2 and FED6 in 3T3-MDR1 but not 3T3 cells (Table 2; Fig 2A), confirming that the lower cytotoxicity of these compounds in 3T3-MDR1 cells was a result of efflux of the compounds via ABCB1. Similarly, there was a 1.7, 3.2 and 6.3 fold increase in GI50 values, for EKB-569, FED2 and FED6, respectively, in the ABCG2 overexpressing cell line (Fig 2B), suggesting that both FED2 and FED6 were effluxed via ABCG2. Pre-treatment with specific ABCG2 inhibitor, FTC, led to a decrease in the GI50 of EKB-569, FED2 and FED6 in MCF7MX but not MCF7 (Table 2, Fig 2B) cells confirming that the differential cytotoxicity of these compounds in the two cell lines were related to efflux via ABCG2.

**Table 2:** The effect of cyanoquinoline inhibitors on growth of ABC transporter-proficient or ABC transporter-deficient cell lines.

Drug (GI50 units)	MCF7		MCF7MX		3T3		3T3-MDR1	
	-FTC	+FTC	-FTC	+FTC	-Zosuquidar	+Zosuquidar	-Zosuquidar	+Zosuquidar
EKB-569 (μM)	2.61±0.07	2.55±0.04	4.28±0.23	1.54±0.06	1.06±0.6	1.15±0.6	5.75±0.7	2.7±0.8
FED2 (μM)	14.9±8.6	14.8±8.54	27.6±15.9	10.3±5.95	7.0±1.2	4.5±0.8	36.0±1.1	5.9±0.6
FED6 (μM)	12.0±6.93	12.3±7.1	75.9±43.8	37.9±21.9	10.7±1.0	7.8±0.7	>100	13.6±0.6
FED14 (μM)	11.4±1.36	11.2±1.11	6.53±0.34	10.1±0.73	14.8±0.7	14.8±0.7	8.7±0.7	12.2±0.6
Vinblastine (nM)	5.12±0.74	5.36±0.63	2.08±0.41	2.66±0.66	1.8±1.0	1.5±1.2	219±0.8	1.1±1.1
Mitoxantrone (μM)	10.7±6.18	6.62±3.82	2500±1440	22.8±13.1	37.0±0.8	197.0±0.6	141±1.0	57±1.0

Data are expressed as 50% growth inhibitory concentration, GI50 ± SE, determined from SRB cell viability assays in MCF7 and MCF7MX with or without 1 h pre-treatment with 10 μM FTC and in 3T3 and 3T3-MDR1 with or without pre-treatment with 0.3 μM Zosuquidar. Vinblastine and Mitoxantrone were used as positive control compounds. FTC and Zosuquidar were used as ABC transporter modulators.

**Fig 3:**
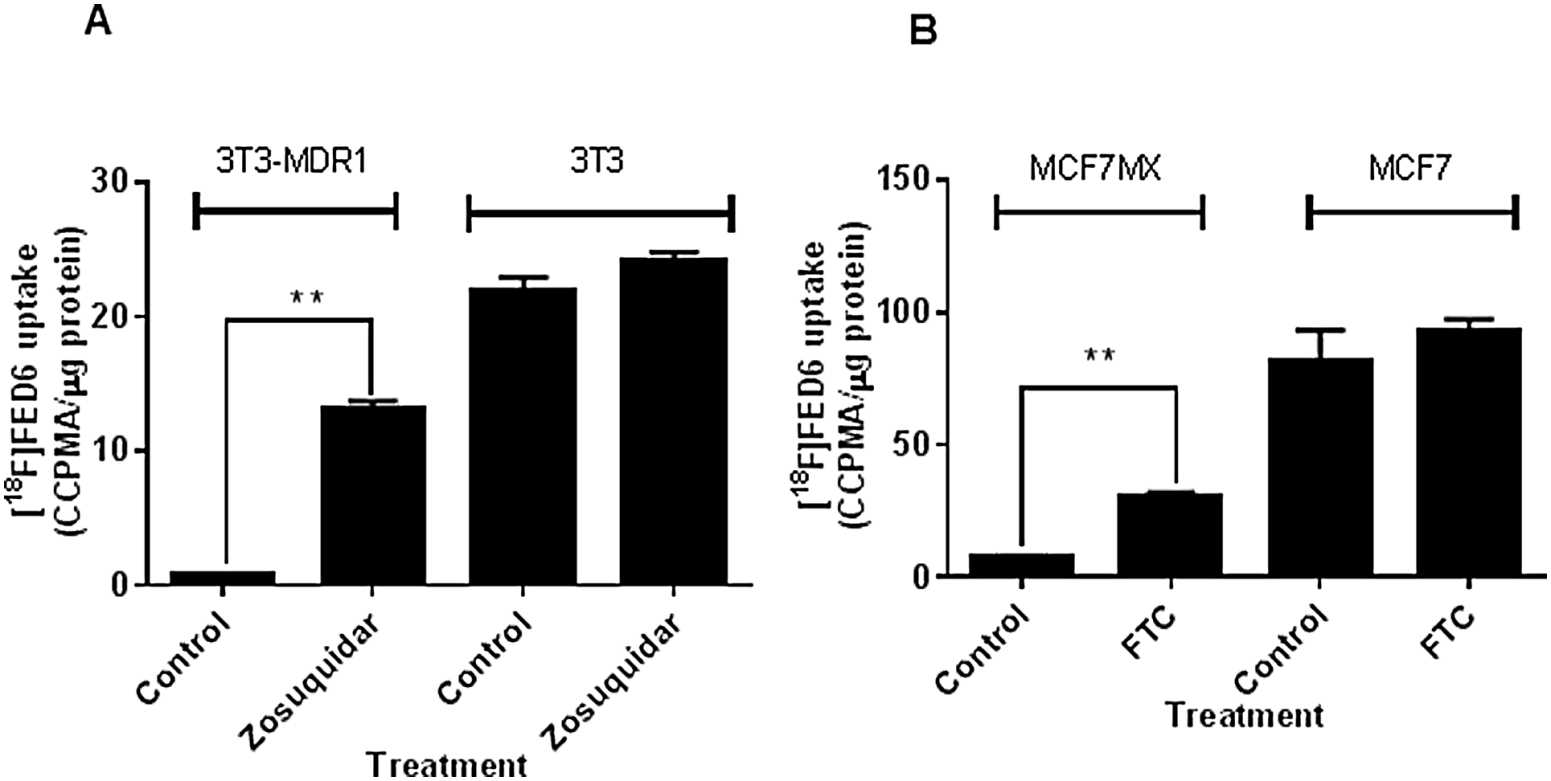
[^18^F]FED6 uptake in ABC transporter expressing cells. A. Protein normalised uptake of [^18^F]FED6 in 3T3 and 3T3-MDR1 cells, following 1 h incubation with 0.22MBq of [^18^F]FED6, with or without pre-treatment with zosuquidar at 0.3 μM for 1 h. B. Protein normalised uptake of [^18^F]FED6 in MCF7 and MCF7MX cells, following 1 h incubation with 0.22MBq of [^18^F]FED6, with or without pre-treatment with 10 μM fumitremorgin C (FTC) for 1 h. Data are mean ± SE of triplicates, repeated twice. Statistical differences between data obtained following an unpaired t-test are indicated on graph; (** = P < 0.01).

To confirm if the GI50 ratio’s above predicted radiotracer efflux, we examined the cell uptake of radiolabelled [^18^F]FED6 in the same pairs of cell lines above expressing different levels of ABC transporters. Expectedly, [^18^F]FED6 uptake was low in ABCB1 and ABCB2 proficient 3T3-MDR1 (0.7 CCPMA/μg of protein) and MCF7MX cells (7.7 CCPMA/μg of protein) compared to isogenic transporter deficient 3T3 (22 CCPMA/μg of protein) and MCF7 cells (81 CCPMA/μg of protein) (Fig 3A and 3B). Furthermore, [^18^F]FED6 uptake increased 19 fold in 3T3-MDR1 cells following zosuquidar pre-treatment and 4 fold in MCF7MX cells following FTC pre-treatment, indicating that ABCB1 or ABCG2 antagonism enhances [^18^F]FED6 uptake.

**Fig 4:**
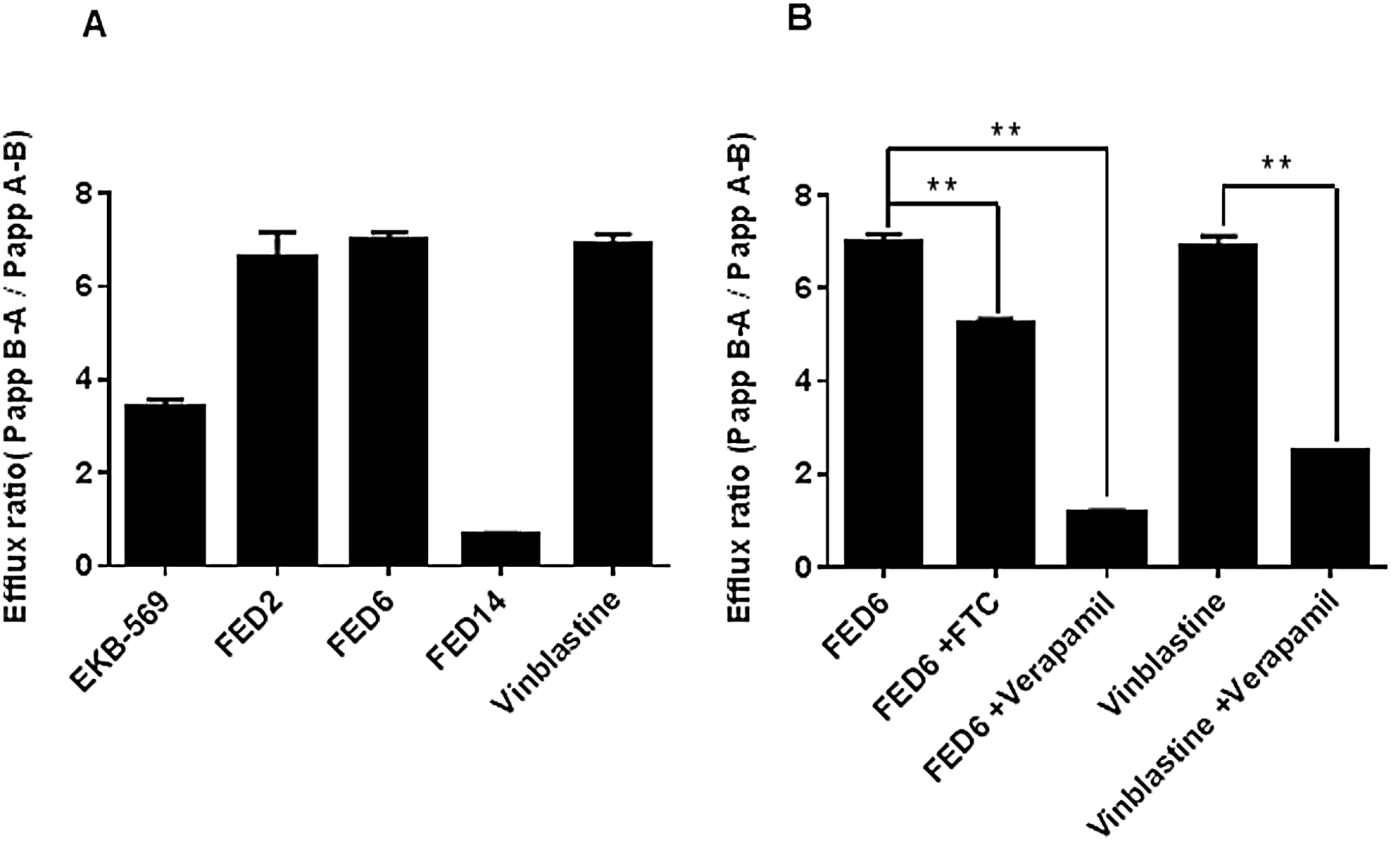
Efflux ratios of cyanoquinoline compounds in caco2 transwell assay. A. Efflux ratios of selected cyanoquinoline compounds. B. Efflux ratios of FED6 and vinblastine with or without pre-treatment with 10 mg/mL of verapamil or 10μM of fumitremorgin C (FTC) for 1 h. All data shows mean ± SE of triplicates, repeated twice. Statistically significant differences between data obtained following an unpaired t-test are indicated on graph; (* = p < 0.05, ** = P < 0.01).

Finally we examined the efflux of cyanoquinoline compounds in the universally exploited caco2 transwell assay. EKB-569, FED2, and FED6 showed efflux ratios >3 (Fig 4A), the accepted threshold for active efflux, indicating active efflux via the ABC transporters [33]. Interestingly, FED14 had an efflux ratio < 1 in keeping with its physicochemical properties (Fig 1). The efflux ratio of FED6 decreased from 6.9 to 1.1 in the presence of ABC transporter inhibitor verapamil further confirming the active efflux of this molecule via the ABC transporters (Fig 4B).

### Higher Affinity of FED6 for Active Mutant EGFR Did Not Translate into Higher [^18^F]FED6 Uptake in Active Mutant EGFR Expressing Cells

**Fig 5:**
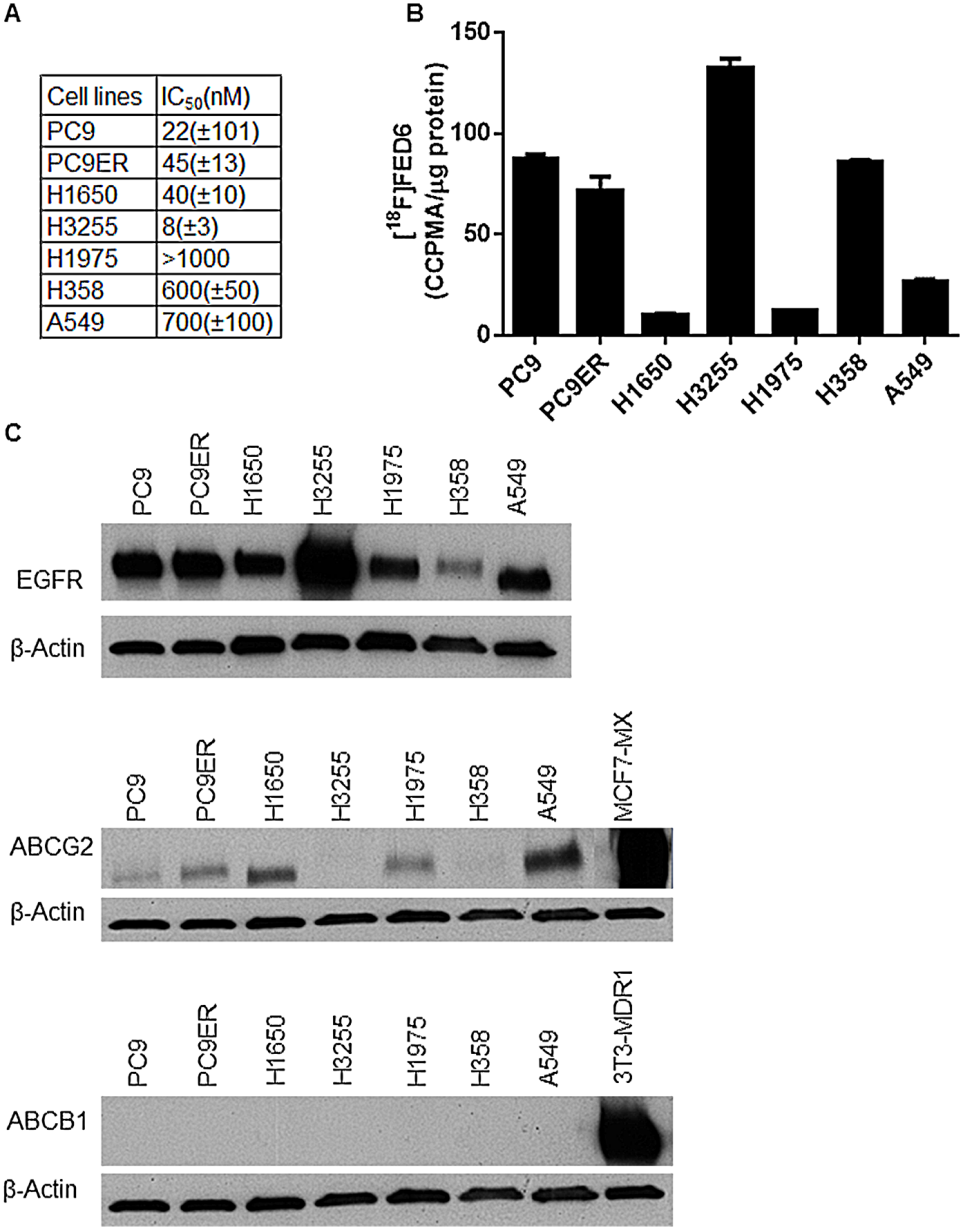
Affinity of FED6 and uptake of [^18^F]FED6 in NSCLC cell lines. A. IC50 values of FED6 obtained from measuring changes in p-EGFR Y1068 in NSCLC cell lines following incubation with FED6 for 3 h. Data are mean IC50 ± SE obtained from three separate experiments. B. Protein normalised uptake of [^18^F]FED6 in NSCLC cell lines following 1 h incubation with 0.22MBq [^18^F]FED6. C. Protein expression of total EGFR, and ABC transporters ABCG2 and ABCB1 in NSCLC cells. MCF7MX and 3T3-MDR1 were used as positive controls for ABCG2 and ABCB1 expression, respectively.

The cyanoquinoline compound FED6 had previously been investigated in cells expressing wildtype EGFR only. We investigated FED6 in the context of mutant EGFR. Inhibition of EGFR phosphorylation (p-EGFR Y1068) by FED6 was measured. The IC50 value for FED6 was significantly lower in active mutant EGFR expressing cell lines at 8–40 nM in H1650 (del 746–750), PC9 (del 746–750) and H3255 (L858R) cells compared to 600–700 nM in wildtype EGFR expressing A549 and H358 cells; H1975 (L858R/T790M) cells had IC50 >1,000 nM (Fig 5A). The *in vitro* derived del 746-750/T790M cell line (PC9ER) showed a 2-fold higher IC50 value in response to FED6 compared to isogenic (PC9) cells devoid of T790M resistance mutation.

The highest cell uptake of [^18^F]FED6 was seen in H3255 cells and lowest in H1650 cells (Fig 5B). Wildtype expressing H358 cells also showed high uptake of [^18^F]FED6 (86 CCPMA/μg of protein). The high affinity of FED6 in H1650 cells (low IC50 value) was discordant with the low [^18^F]FED6 uptake in these cells; similarly, the low affinity of FED6 for wildtype EGFR (high IC50 value) in H358 cells was discordant with high uptake of [^18^F]FED6 in these cells.

EGFR, ABCB1 and ABCG2 protein expression in these cells (Fig 5C) indicated that a lack of correlation between the affinity of FED6 for the different mutant forms of EGFR and the degree of cell uptake of [^18^F]FED6 could be a result of differential expression of EGFR and ABCG2. Indeed, H3255 cells express significantly higher levels of EGFR than the other cell lines which could explain the highest uptake of [^18^F]FED6 in these cells. The lack of ABCG2 in H3255 and H358 could also explain why the uptake of [^18^F]FED6 was highest in these two cell lines regardless of the EGFR mutational status. As ABCB1 protein expression was not detected in any of the cell lines studied we focused on the possibility of overcoming the efflux of [^18^F]FED6 via ABCG2.

### Can ABCG2 Knockdown or Inhibition Overcome Reduced Cell Uptake of Radiolabelled [^18^F]FED6?

**Fig 6:**
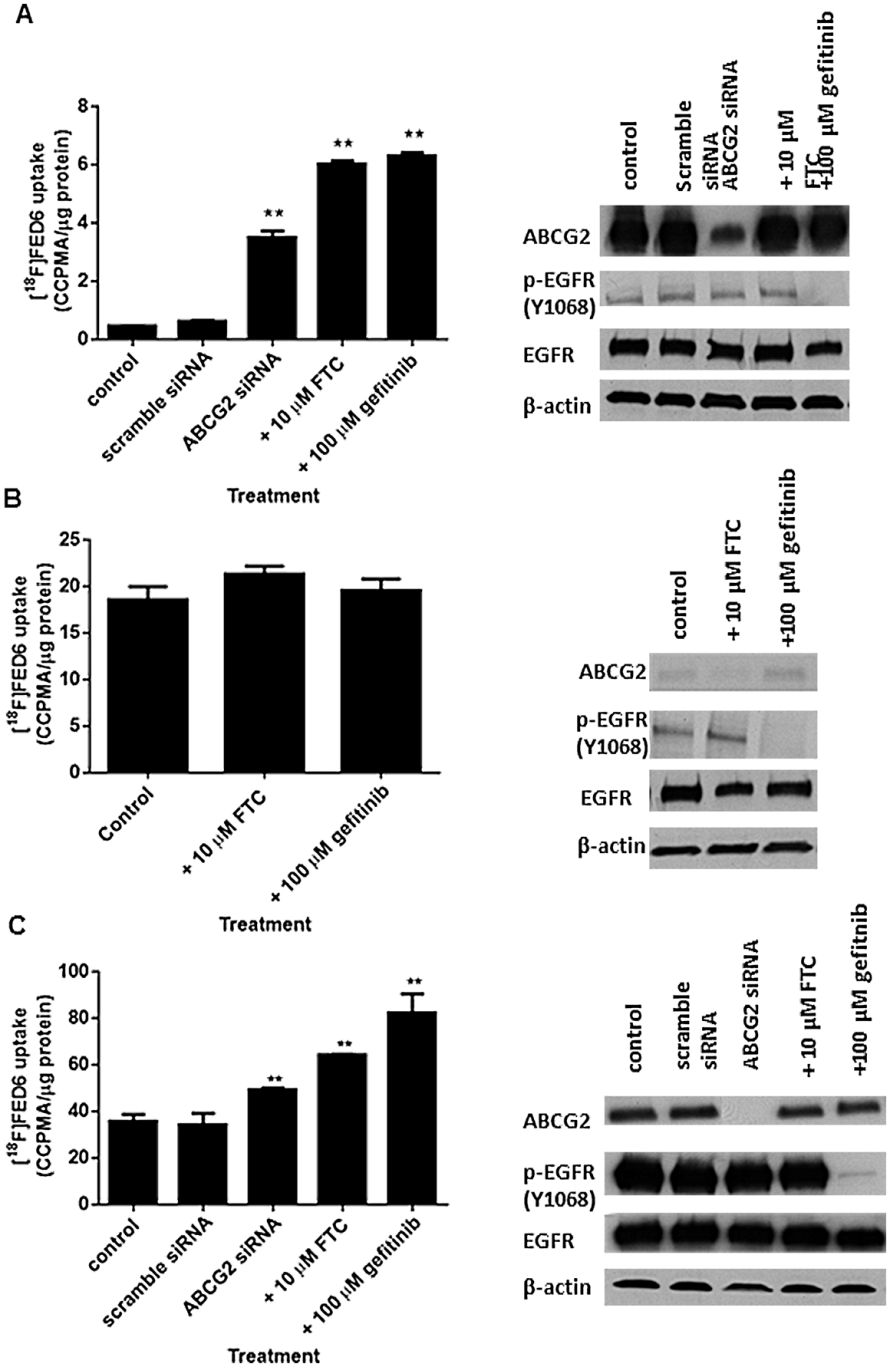
Modulation of [^18^F]FED6 uptake following ABCG2 knockdown or drug inhibition. Protein normalised uptake of [^18^F]FED6 and corresponding protein expression by western blot in A. MCF7MX, B. MCF7, and C. A431 following 1 h incubation with 0.22MBq [^18^F]FED6. MCF7MX and A431 cells were transfected with 25 nM ABCG2- or scrambled-siRNA for 72 h prior to cell uptake. Non-transfected cells were pre-treated with or without 10 μM fumitremorgin C (FTC) or 10 μM gefitinib for 1 h. Data are mean ± SE from triplicates, repeated twice. β-actin was used as a loading control. (** = P < 0.01).

One way of simplifying EGFR-specific tracer uptake interpretation is to remove the contribution of ABCG2 activity. Thus, we examined the specific role of ABCG2 in the efflux of [^18^F]FED6 in MCF7MX cells (+++ ABCG2; + EGFR and p-EGFR expression) following ABCG2 siRNA transfection or pre-treatment with ABCG2 inhibitors, FTC and gefitinib. The effect of FTC (positive control) and gefitinib were examined in isogenic ABCG2 negative MCF7 cells. [^18^F]FED6 uptake increased from 0.6 CCPMA/μg protein in scramble siRNA transfected MCF7MX cells to 3.5, 6 and 6.3 following pre-treatment with ABCG2 siRNA, FTC (positive control) and gefitinib, respectively (Fig 6A). The lower [^18^F]FED6 uptake following ABCG2 siRNA pre-treatment compared to FTC or gefitinib could be due to incomplete knockdown of ABCG2. The cell uptake of [^18^F]FED6 was unchanged following pre-treatment with FTC or gefitinib in ABCG2 negative MCF7 cells (Fig 6B). To further scrutinize the specificity of this approach, we repeated the study in an independent cell line, A431 (+ ABCG2; +++ EGFR and p-EGFR expression). The high unmodulated [^18^F]FED6 uptake at baseline could be explained in part by high EGFR expression (Fig 6C). ABCG2 knockdown was near complete and was associated with increased [^18^F]FED6 uptake. FTC and gefitinib again increased [^18^F]FED6 uptake in this cell line.

### Evaluation of a Hydrophilic Cyanoquinoline, FED20

While it is feasible to enhance radiotracer uptake via inhibition of ABCG2, an ultimate goal for this class of radiotracers is to design out substrate specificity for ABC transporters. As well as being a substrate for ABCB1 and ABCG2, FED6 is also associated with high non-specific binding. The high lipophilicity (Log*P*) of FED6 (3.85) (Fig 1) is thought to contribute at least in part to this property. We recently reported the synthesis of a glycosylated cyanoquinoline derivative, FED20, designed to reduce the lipophilicity [31]. The Log*P* of FED20 was significantly lower than that of FED6 at 2.51, while retaining affinity for EGFR; IC50 for inhibition of cell-free EGFR phosphorylation was 4.5 nM compared to 1.8 nM for FED6 [18,31]. While features such as MW, PSA, and n(N+O) were still suggested of active efflux, we predicted that FED20 would have improved uptake selectivity profile due to the lower Log*P* [34].

**Fig 7:**
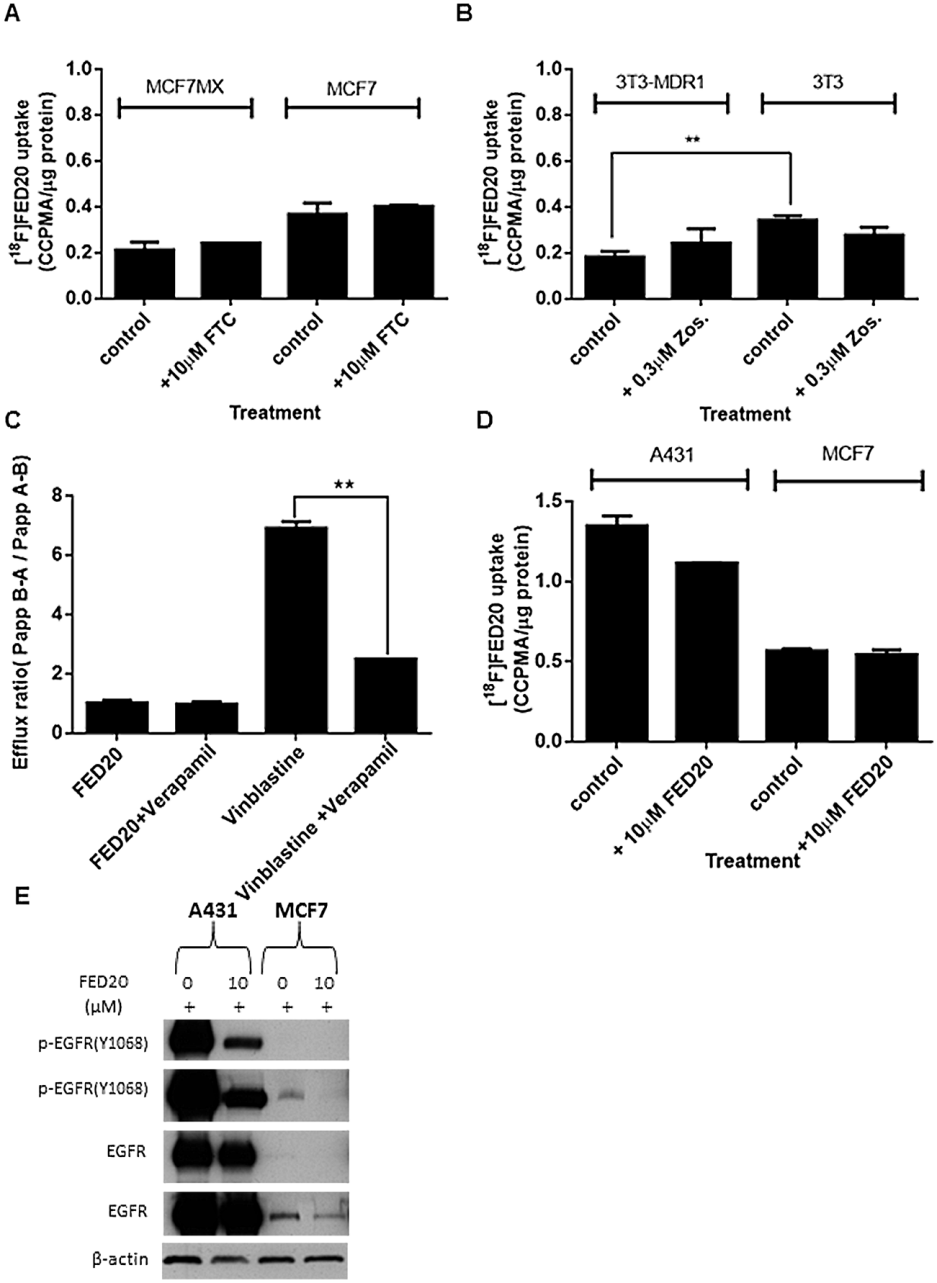
Cell uptake of [^18^F]FED20 in relation to ABC transporter substrate specificity and specificity for EGFR. Protein normalised uptake of [^18^F]FED20 in A. MCF7 and MCF7MX, or B. 3T3 and 3T3-MDR1 following 1 h incubation with 0.55 MBq of FED20 with or without pre-treatment with 10 μM fumitremorgin C (FTC) or 0.3 μM zosuquidar (zos) for 1 h. C. Efflux ratio of FED20 and vinblastine from a caco2 transwell assay with or without 1 h pre-treatment with 10 mg/ml of verapamil. D. Protein normalised uptake of [^18^F]FED20 in A431 and MCF7 cells following 1 h incubation with 0.55MBq of [^18^F]FED20 with or without pre-treatment with 10 μM FED20. Data are mean ± SE of triplicate, repeated twice. E. Protein expression of p-EGFR Y1068 and EGFR in A431 and MCF7 cells. (** = P < 0.01).

A number of observations indicated that FED20 was not a substrate for ABCB1 or ABCG2 transporters: a) In the MCF7/MCFMX and 3T3/3T3-MDR isogenic cell lines, no biologically significant differences in uptake of radiolabelled FED20 was seen (Fig 7A and 7B). Furthermore, pre-treatment with specific transporter inhibitors, FTC in the MCF7/MCF7MX cells and zosuquidar in the 3T3/3T3-MDR1 cells, did not lead to an increase in the uptake of [^18^F]FED20 in the ABCG2 or ABCB1 expressing cell lines. b) FED20 was not actively effluxed by caco-2 monolayers, with efflux ratio close to unity (1.3) (Fig 7C). In addition, pre-treatment with zosuquidar did not have any effect on the permeability ratio. In contrast, the permeability ratios of vinblastine (positive control), a substrate of ABC transporters, and FED6 (Fig 4) were greater than 3, the accepted threshold for active efflux; pre-treatment with zosuquidar reduced the ratio for vinblastine from 6.7 to 1.7 (Fig 4). Expectedly, [^18^F]FED20 uptake was higher in A431 cells compared to EGFR low MCF7 (Fig 7D and 7E). Pre-treatment with unlabelled FED20 decreased [^18^F]FED20 uptake in the former but not in the latter cell line. Notably, the lower overall uptake selectivity of [^18^F]FED20 in cells could be explained by the hydrophilic nature of the radiotracer.

**Fig 8:**
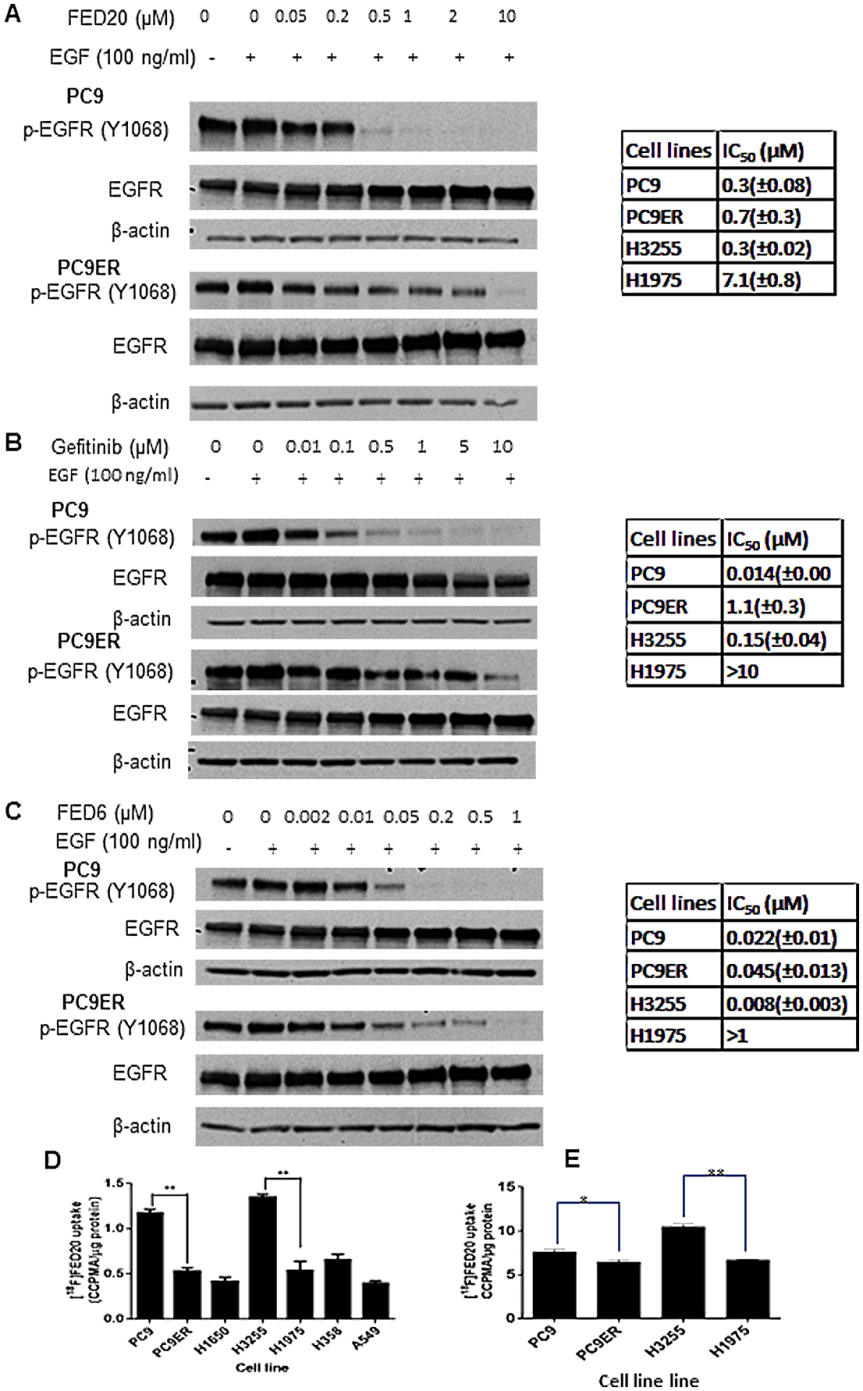
Comparative affinities FED20, gefitinib and FED6 in TKI sensitive and resistant NSCLC cell lines and [^18^F]FED20 uptake in the cell lines. Typical of p-EGFR Y1068 and EGFR protein expression following 3 h incubation with increasing concentrations of A. FED20, B. gefitinib, or C. FED6 in PC9 and PC9ER cells; summary IC50 values for all cell lines determined from densitometry analysis of p-EGFR Y1068 in the western blots are shown. D. Protein normalised uptake of [^18^F]FED20 in TKI sensitive and resistant cells following 1 h incubation with 0.55 MBq of [^18^F]FED20 (low specific activity; 7.3 GBq/μmol). E. Protein normalised uptake of [^18^F]FED20 in TKI sensitive and resistant cells following 1 h incubation with 0.74 MBq of [^18^F]FED20 (higher specific activity; 52 GBq/μmol). (* = p < 0.05, ** = P < 0.01).

Finally, we examined the utility of [^18^F]FED20 for discriminating isogenic mutant EGFR cell lines that expressed del E746-A750 EGFR (PC9) or del E746-A750/T790M (PC9ER) for which [^18^F]FED6 was not discriminatory. Both cell lines expressed similar levels of EGFR, however, the IC50 for inhibition of EGFR phosphorylation was 2-fold higher in PC9ER (0.7μM) compared to PC9 cells (0.3μM) (Fig 8A–8C). The same trend of higher potency in PC9 versus PC9ER was seen with gefitinib and FED6 (positive controls) although the magnitude of reduction was higher for gefitinib and FED6. In keeping with this observation, the uptake of [^18^F]FED20 was higher in the PC9 compared to PC9ER cells (Fig 8D). The overall lower potency for inhibition of p-EGFR inhibition is also likely due to the hydrophilic character of the compound. [^18^F]FED20 uptake was also low in cells harboring EGFR wildtype or T790M mutation. The H1650 cell line was unique in that while it harbors del E746-A750 active mutant EGFR, it showed low [^18^F]FED20 uptake. The absolute uptake of [^18^F]FED20 was improved at higher specific radioactivity (Fig 8E).

## Discussion

EGFR remains an important transmembrane receptor target in cancer and molecular imaging of EGFR status—expression or mutation—using PET could aid patient selection for treatment with novel reversible or irreversible inhibitors. A key threat to developing successful imaging agents is existence and evolution of multidrug resistance phenotype. Starting from the ABC transporter substrate [^18^F]FED6, we describe a novel cyanoquinoline radiotracer [^18^F]FED20 that is devoid of ABC transporter activity.

One of the key components of MDR is the increase in expression of ABC transporters [35,36]. In the context of PET imaging, ABC transporter substrate specificity is also an important mechanism of radioprobe clearance. Indeed, as only tracer amounts of the radiolabelled probe are injected into the patients, efflux via such transporters will significantly decrease the potential accumulation of the radiotracer in the tumour. Several TKIs, including EKB-569 from which the FED series of compounds are derived have been shown to be substrates for members of the ABC transporter family [24]. The initial *in silico* characterisation of the FED series of compounds, indicated that these compounds had several characteristics which make them potential substrates for both ABCB1 and ABCG2 transporters: MW above 400 Da, Log*P* > 3 (ABCB1 substrate) and PSA>85Å^2^ (ABCG2 substrate). [37,38]. We therefore predicted that FED6, FED2, and FED9 would be substrates for ABCB1 and ABCG2, whereas FED14 that had a lower MW would not. The cell viability assays performed in the paired cells lines that are proficient or deficient for specific ABC transporters, allowed us to indirectly verify this prediction. The higher GI50 values of FED6 in both the ABCB1 expressing 3T3-MDR1 cells and the ABCG2 expressing MCF7MX cells compared to the paired matched cells that expressed no transporters, as well as decreases in GI50 when the transporters were inhibited with zosuquidar or FTC indicated substrate specificity of FED6 for both transporters. By measuring efflux ratios, corresponding to the ratio between the rate of secretion over the rate of absorption, we determined that FED6, together with EKB-569, and FED2 were indeed effluxed by the physiologically relevant differentiated caco2 cell model; efflux ratios above 1 were indicative of active ABC transporter efflux [33]. The efflux of FED6 was reduced in the presence of ABC transporter inhibitor verapamil further confirming the active efflux of FED6. The indirect measurement of cytotoxicity in paired cells, compound efflux in caco2 and modulation by pharmacological inhibitors translated into predicted differences in [^18^F]FED6 uptake in the ABC transporter proficient versus deficient cells.

EGFR is overexpressed in several cancers including, breast, ovarian, brain and colorectal [39], and this has led to development of specific TKIs directed against EGFR. However, efficacy of gefitinib and erlotinib against wildtype EGFR in patients was disappointing, with only a small fraction of unselected patients responding to TKI treatment [40]. The subgroup of patients, shown to express active mutant EGFR, had significantly higher response rates to TKI treatment [10]. Identifying patients that express mutant EGFR, non-invasively via PET imaging could assist in therapeutic selection. To date no PET tracers have been approved for the imaging of either wildtype or mutant EGFR in the clinic. The discovery of [^18^F]PEG6-IPQA, an L858R mutant EGFR specific [^18^F] PET radiotracer that showed a 7.6 fold higher uptake in H3255 (L858REGFR) compared to H1975 (L858R/T790M EGFR) xenografts was an important breakthrough in the imaging of EGFR [19]. This radiotracer was only studied in L858R mutant EGFR and further investigation in other common active mutant EGFR, including del E746-A750 EGFR would be important. Furthermore, tumour uptake of the most recent iteration of irreversible EGFR imaging agents, [^18^F]afatinib, was sensitive to modulation by the ABCB1-specific inhibitor, tariquidar.

We investigated [^18^F]FED6 for the imaging of mutant EGFR. FED6 was more potent against active mutant EGFR expressing cells than wildtype or resistant mutant EGFR expressing cells with respect to inhibition of p-EGFR. The uptake of [^18^F]FED6 was high in H3255 (L858R EGFR) and the PC9 (del E746-A750) cells, and lowest in gefitinib resistant H1650 (del 746–750) and H1975 cells (L858R/T790M) cells. The higher uptake of [^18^F]FED6 in H3255 cells as opposed to the low uptake in H1975 cells correlated with the differential affinity of FED6 in these two cells lines, assessed by western blot. However, it is important to note that expression levels of EGFR as well as mutant status could impact on the uptake of [^18^F]FED6. H3255 cells had the highest expression of EGFR compared to the other NSCLC cell lines investigated. We cannot claim that the higher uptake of [^18^F]FED6 in H3255 cells is a consequence of the higher affinity of FED6 for L858R mutant EGFR since it could also be due to higher expression of EGFR or a combination of these. We addressed this conundrum by assessing uptake of [^18^F]FED6 in isogenic PC9 and PC9ER cell lines that have the same levels of EGFR protein expression but differ in mutational status; no statistical difference in the uptake of [^18^F]FED6 was seen. Furthermore, the low uptake of [^18^F]FED6 in H1650 cells was at odds the potent reduction of p-EGFR by FED6 in these cells. Notably, ABCB1 was not expressed in any of the cell lines studied, whereas ABCG2 was expressed in all the cell lines with the exception of the H3255 and H358 cells. Therefore the higher uptake of [^18^F]FED6 in H3255 and H358 compared to the low uptake in A549 and H1650 could also be related to expression of ABCG2.

As only ABCG2 was expressed in the cell lines used, we wondered whether it would be feasible to improve EGFR detection properties of [^18^F]FED6 by blocking this transporter using siRNA or pharmacological inhibitors FTC and gefitinib [41–44]. Indeed, both siRNA and pharmacological inhibition of ABCG2 increased [^18^F]FED6 uptake with magnitude depending on ABCG2 expression/knockdown and EGFR expression. The uptake of [^18^F]FED6 following inhibitor pre-treatment was consistently higher than following ABCG2 siRNA transfection which could be due to more efficient inhibition by the compounds. Rather than having to block the effect of the ABC transporters to which [^18^F]FED6 is a substrate, designing a radiotracer than lacks the ABC transporter substrate specificity is preferred.

We have recently reported a hydrophilic cyanoquinoline compound FED20 [31]. The addition of a glucose moiety to form FED20 reduced Log*P* (to 2.1) compared to that of FED6 (3.85). The higher uptake of [^18^F]FED20 in the A431 versus MCF7 cells was indicative of the specificity of the tracer for EGFR. Furthermore, the uptake of [^18^F]FED20 in isogenic ABCB1 and ABCG2 proficient or deficient cell lines, supported by the unremarkable efflux characteristics of unlabelled FED20 by differentiated caco2 cells indicated that the compound lacked substrate specificity for ABCB1 and ABCG2. We have not tested the impact of all known ABC transporters in this study. For example, we did not directly test the impact of ABCC1 (MRP1) expressed in NSCLC as a potential risk for interaction with FED6. Even though no isogenic cell line information was generated for MRP1, the caco-2 data are broadly suggestive of a lack of interaction. Furthermore, verapamil is a dual ABCB1-MRP1 inhibitor, thus, a lack of interaction could be inferred from the verapamil inhibitor data [45,46]. The magnitude of [^18^F]FED20 uptake was > 5-fold lower than that seen for [^18^F]FED6. Despite its lower lipophilicity, this result is counterintuitive given the lack of transporter activity. The lower potency of FED20 on p-EGFR compared to FED6 suggests that the lower lipophilicity of FED20 may somewhat compromise its cellular delivery and engagement of target. An additional potential reason for the low [^18^F]FED20 uptake is lower specific activity (7.3 GBq/μM) of the reported radiosynthetic method [31] compared to [^18^F]FED6 (48 GBq/μmol) with some self-blocking by cold compound being a possibility. In keeping with this hypothesis, absolute uptake of [^18^F]FED20 increased at higher specific radioactivity (52 GBq/μmol) although the difference in uptake between PC9 and PC9ER was less (albeit significant) than seen under low specific activity conditions.

In view of the lack of ABC transporter substrate specificity, we investigated the potential of this tracer for the imaging of mutant versus wild type EGFR. We initially focused on two cell lines with nearly equivalent EGFR expression and for which [^18^F]FED6 uptake was non-discriminatory; PC9 cells expressing active mutant EGFR had a 2-fold higher uptake of [^18^F]FED20 compared to PC9ER cells. [^18^F]FED20 uptake was also low in other cells harboring EGFR wildtype or T790M mutation except the H1650 cell line, which while harboring a del E746-A750 active mutant EGFR, showed low [^18^F]FED20 uptake. Despite harboring active mutant EGFR, this cell line is known to be resistant to gefitinib. PTEN loss has been proposed as a key genomic resistance mechanism in the cell line [47], but it is unclear if this alteration affects cellular ATP levels or ATP affinity of the EGFR receptor in addition to the known activity of PTEN loss/AKT activation in cell survival.

So far we have demonstrated that [^18^F]FED20 has useful properties for discriminating EGFR inhibitor resistance (genomic or functional) in NSCLC cell lines. Further work—in particular metabolism, biodistribution and imaging contrast—is required to advance [^18^F]FED20 towards imaging in humans for upfront detection of mutational status, monitoring response to treatment, and assessing the development of drug resistance [16,48]. The current recommendations are that, NSCLC patients undergo screening for mutations to EGFR before the start of therapy [49]. This allows the selection of patients with active mutant to be given TKI gefitinib or erlotinib. Indeed gefitinib was approved in the UK by NICE in 2009 for first line treatment for advanced metastatic NSCLC with active mutant EGFR [50]. PET imaging of active mutant EGFR could complement this screening as biopsies are not always possible and different nodules may present different mutational expression that would not be picked up if one site was biopsied [51]. PET may also be useful in detecting secondary resistance.

### Conclusion

We demonstrated the role of the substrate specificity of the cyanoquinoline compound FED6 in the low accumulation of the radiotracers in the tumour. Blocking ABCG2 with gefitinib led to an increase in [^18^F]FED6 uptake. FED20, which lacked substrate specificity for ABCB1 and ABCG2 was found to have favourable properties for detection of EGFR resistance.
